# Consensus Recommendations for Nutritional Intervention in Pediatric Oncology (Ages 4–18 Years) on Behalf of the Romanian Society of Pediatric Hematology and Oncology and the Romanian Society of Pediatric Gastroenterology, Hepatology and Nutrition

**DOI:** 10.3390/nu18121889

**Published:** 2026-06-11

**Authors:** Irina Avrămescu, Steluța Boroghină, Alexandru Pârvan, Laura Bălănescu, Cecilia Negrei, Elena Albu, Cristina Georgiana Jercan, Andra Daniela Marcu, Horațiu Albu, Anca Coliță

**Affiliations:** 1 Faculty of Medicine, University of Medicine and Pharmacy Carol Davila, 050474 Bucharest, Romania; irina.avramescu@drd.umfcd.ro (I.A.); laura.balanescu@umfcd.ro (L.B.); cristina.jercan@umfcd.ro (C.G.J.); andra.marcu@umfcd.ro (A.D.M.); anca.colita@umfcd.ro (A.C.); 2 Department of Pediatrics, and Bone Marrow Transplantation Unit, Fundeni Clinical Institute, 022328 Bucharest, Romania; 3 Romanian Society of Pediatric Hematology and Oncology, 022328 Bucharest, Romania; cecilia.negrei@gmail.com; 4 Department of Pediatrics, Fundeni Clinical Institute, 022328 Bucharest, Romania; 5 Romanian Society of Pediatric Gastroenterology, Hepatology and Nutrition, 400177 Cluj-Napoca, Romania; alexandru.pirvan@umfcluj.ro; 6 Faculty of Medicine, University of Medicine and Pharmacy Iuliu Hațieganu, 400012 Cluj-Napoca, Romania; 7 Department of Pediatrics, Emergency Clinical Hospital for Children, 400001 Cluj-Napoca, Romania; 8 Department of Pediatric Surgery, Grigore Alexandrescu Children’s Emergency Clinical Hospital, 011743 Bucharest, Romania; 9 Romanian Society of Pediatric Surgery, 500058 Brașov, Romania; 10 MedEuropa, Radiotherapy and Medical Oncology, 022343 Bucharest, Romania; 11 Center for Dietetics, Oncology Nutrition, and Integrative Medicine, 013307 Bucharest, Romania; elena.albu@naturomedica.ro (E.A.); dr.albu@naturomedica.ro (H.A.)

**Keywords:** pediatric oncology, nutritional support, malnutrition, Delphi consensus, enteral nutrition, parenteral nutrition, nutritional screening, clinical nutrition guidelines, cancer survivors, palliative care

## Abstract

Background: Malnutrition, encompassing both undernutrition and overnutrition, is a common complication in children with cancer and is associated with impaired treatment tolerance, increased infection risk, altered pharmacokinetics, reduced quality of life, and poorer survival outcomes. Despite its importance, nutritional management in pediatric oncology lacks a unified, systematically organized clinical framework applicable to the full trajectory of the disease. Objective: This study aimed to develop expert consensus recommendations for nutritional intervention in pediatric oncology patients aged 4 to 18 years. Methods: A modified electronic Delphi (e-Delphi) process was conducted with a multidisciplinary expert panel of 22 specialists, including pediatric oncologists, pediatric gastroenterologists, clinical nutrition specialists, radiotherapy specialists, and pediatric surgeons. Statements were rated on a 9-point Likert scale across two anonymous rounds, with consensus predefined as ≥80% agreement. Results: Forty-one consensus recommendations were formulated across nine domains: nutritional screening and assessment, energy and protein requirements, micronutrient supplementation, physical activity, nutritional support escalation, refeeding syndrome prevention, treatment-specific management, survivorship, and palliative care. All recommendations achieved the predefined consensus threshold. Conclusions: This Delphi consensus provides a structured, multidisciplinary, and clinically actionable framework for nutritional management across the full trajectory of childhood cancer and is intended to reduce institutional variability and improve patient outcomes.

## 1. Introduction

The remarkable progress achieved over recent decades in the treatment of pediatric cancer is attributable not only to the optimization of antineoplastic therapies but also to substantial advances in supportive care, among which clinical nutrition plays an important role. Nutritional status influences treatment tolerance, complication rates, length of hospitalization, survival outcomes, and quality of life, both in the short and long term [1,2].

In Romania, pediatric oncology lacks a standardized framework adapted to local resources for the nutritional assessment and management of children and adolescents with cancer. While recent international efforts have made significant progress in addressing nutritional care gaps in resource-limited settings [3], a comprehensive framework adapted to the clinical complexity and resource availability of European pediatric oncology centers remains lacking. This document addresses this gap by proposing consensus recommendations based on current scientific evidence and collective clinical experience.

## 2. Materials and Methods

### 2.1. Study Design

This study was designed as a modified electronic Delphi (e-Delphi) process for developing consensus recommendations for nutritional intervention in pediatric oncology patients. The Delphi methodology was selected to ensure structured, anonymous, and iterative consensus-building among multidisciplinary experts. The study was conducted between February 2026 and March 2026.

### 2.2. Panel Selection

A multidisciplinary expert panel comprising 22 specialists was convened. The panel included fifteen pediatricians and pediatric oncologists, each with more than 15 years of experience in pediatric oncology, four pediatric gastroenterologists, each with more than 15 years of clinical experience, one radiotherapist with 10 years of pediatric oncology experience, one pediatric surgeon with more than 20 years of experience and one physician specializing in oncology nutrition with 10 years of experience in the field. Panel members represented institutions across Romania, including Bucharest, Cluj-Napoca, Iași, Timișoara, and Constanța, and they were affiliated with three national societies in Romania (Romanian Society of Pediatric Hematology, Romanian Society of Pediatric Gastroenterology, Hepatology and Nutrition, and Romanian Society of Pediatric Surgery). All panel members were involved in the management of pediatric oncology patients and had recognized clinical and/or academic expertise in their fields. Experts were invited by direct email communication. Participation was voluntary and uncompensated. All experts confirmed their agreement to participate prior to Round 1.

### 2.3. Development of Initial Statements

An initial set of 41 statements was developed by the scientific committee through a review of current international guidelines, including those published by ESPEN, ESPGHAN, SIOP, and the Children’s Oncology Group (COG), combined with a review of meta-analyses, systematic reviews, and recent clinical studies. Statements were additionally shaped by the identification of gaps in nutritional practice within the local institutional context. Statements were organized into the following domains: (1) nutritional screening and assessment of nutritional status; (2) energy and protein requirements; (3) micronutrient management; (4) physical activity; (5) oral, enteral, and parenteral nutritional interventions; (6) prevention of refeeding syndrome; (7) nutritional management during chemotherapy, radiotherapy, hematopoietic stem cell transplantation, and surgery; (8) survivorship; and (9) palliative care.

### 2.4. Delphi Process

The consensus process consisted of two anonymous rounds conducted electronically via Google Forms (Google LLC, Mountain View, CA, USA).

Round 1. Experts rated each statement on a 9-point Likert scale (1 = strongly disagree, 9 = strongly agree). Ratings of 7–9 were considered indicative of agreement. Participants were also invited to provide comments, suggest modifications, and propose additional statements. Round 1 was open for 2 weeks, from 16 February to 1 March 2026. The response rate in Round 1 was 100%. Consensus was predefined as agreement by ≥80% of responding experts. Following Round 1, responses were analyzed quantitatively. Statements that met the predefined threshold were kept without modification. Statements not reaching consensus were reviewed considering anonymized expert feedback; 6 statements were revised accordingly. A summary of Round 1 results, including the distribution of ratings and anonymized comments, was provided to all panelists before Round 2.

Round 2. A revised questionnaire was distributed to the panel. Experts were asked to re-evaluate modified statements considering the Round 1 feedback. Round 2 was open for 2 weeks, from 9 March to 22 March 2026. The response rate in Round 2 was 100%. Consensus was achieved for all remaining statements during Round 2.

### 2.5. Definition of Recommendation Strength

The strength of each recommendation was determined based on the level of agreement among experts, the quality and consistency of scientific evidence, and clinical applicability. The wording reflected the level of confidence. “We recommend” was used for strong consensus (≥90% agreement) and high-quality or consistent levels of evidence; “We suggest” was used for recommendations supported by moderate consensus (80–89% agreement) and/or lower levels of evidence; and “May be considered” was used for exploratory recommendations based on emerging or preliminary data.

### 2.6. Ethical Considerations

The study involved healthcare professionals and did not include patient data, patient-identifiable information, or interventions involving human subjects. Participation was voluntary, and responses were anonymized. According to the institutional policy of Carol Davila University of Medicine and Pharmacy, Bucharest, formal ethics committee approval was not required for expert consensus surveys. Artificial intelligence-assisted tools were used for language editing and stylistic refinement of the manuscript. The authors take full responsibility for the accuracy, integrity, and originality of the final content.

## 3. Results

Forty-one key questions were identified and addressed by the expert panel and are presented in a Question-and-Answer form.

The authors formulated a total of 41 consensus recommendations addressing key aspects of nutritional management in pediatric oncology patients. These recommendations cover nutritional screening and assessment, identification of patients at risk of malnutrition, determination of energy and protein requirements, selection and escalation of nutritional support (oral, enteral, and parenteral), prevention and management of refeeding syndrome, and nutritional strategies during active anticancer therapies, survivorship, and palliative care. The recommendations are detailed in the subsequent sections, together with their scientific justification.

### 3.1. Nutritional Screening and Evaluation of Nutritional Status

Nutritional status significantly influences treatment tolerance, complication rates, and overall outcomes in pediatric oncology patients. Early implementation of nutritional screening, comprehensive nutritional assessment and individualized support facilitates timely identification of nutritional risk and appropriate intervention. Continuous monitoring throughout treatment and survivorship is essential, as nutritional requirements may change due to treatment-related toxicities and disease evolution [2,4].

Within the context of periodic nutritional evaluation in pediatric oncology, both initial screening and detailed nutritional assessment are best conducted by trained professionals who should ensure longitudinal follow-up throughout the entire treatment period and into survivorship. Close collaboration among physicians specializing in oncology nutrition, dietitians, clinical nutrition specialists, and pediatric oncologists is essential to provide individualized and optimized nutritional care for children diagnosed with cancer [2].

At the time of diagnosis, certain factors associated with an increased risk of treatment-related malnutrition may already be identified. Their presence requires closer surveillance and more frequent nutritional screening. Risk factors include: age below 1 year; pre-existing malnutrition at diagnosis; tumors involving the abdomen, head and neck, or central nervous system; high-risk disease and advanced stages, including metastatic, recurrent and/or progressive cancer; persistent nausea, vomiting, diarrhea, constipation, dysphagia, odynophagia, anorexia, or gastrointestinal tract obstruction secondary to tumor compression or infiltration; planned hematopoietic stem cell transplantation (HSCT); anticipated long-term corticosteroid therapy; planned radiotherapy for the abdominal and/or pelvic or head and neck regions; significant comorbidities; low socioeconomic status; and limited adherence or support from family/caregivers [5].

A comprehensive nutritional evaluation may be structured according to the A–B–C–D framework:

A (Anthropometry), B (Biochemistry), C (Clinical evaluation), D (Dietary assessment)

A. Anthropometry should include body weight and height, body mass index (BMI) and mid-upper arm circumference (MUAC), which has been shown to be a reliable indicator of nutritional status [6] and correlates with muscle mass. Replacing MUAC with the combination of MUAC and triceps skinfold thickness (TSFT) allows for a more refined evaluation, as these parameters are less influenced by tumor mass and provide information on both lean body mass and fat mass [7].

Given that children undergoing treatment for all types of cancer often demonstrate significantly reduced lean body mass and increased fat mass compared with healthy controls, bioelectrical impedance analysis (BIA) may be considered an objective, reliable, and reproducible method for longitudinal assessment [8].

Where available, BIA may be used as an alternative to MUAC or MUAC combined with TSFT.

B. Biochemistry should be interpreted with caution, as laboratory parameters may be altered by the malignancy itself and/or anticancer therapies. Nonetheless, appropriate investigations are necessary to evaluate protein status, organ function, calcium–phosphorus metabolism and bone health, the presence and degree of systemic inflammation, and specific vitamin and micronutrient deficiencies [2].
C. Clinical evaluation aims to identify physical signs of malnutrition as well as symptoms, functional impairments, or comorbidities that may negatively affect oral intake.
D. Dietary assessment should ideally be performed by a registered dietitian, who should subsequently provide longitudinal follow-up throughout treatment and survivorship [2]. Dietary evaluation is valuable for estimating daily calorie intake and for screening potential micronutrient deficiencies [7]. As part of a comprehensive evaluation of nutritional status, a detailed dietary history should also be obtained. This should include macro- and micronutrient intake, food aversions, allergies or intolerances, current eating patterns, family-related feeding behaviors, and household food hygiene practices [2]. Food security considerations should not be overlooked.

Malnutrition in pediatric oncology patients should be classified according to severity, as this directly guides the urgency and intensity of nutritional intervention. Based on internationally recognized criteria from WHO and ESPEN [9,10], the following thresholds are recommended for clinical use:

- Mild malnutrition is defined as a BMI-for-age z-score between −1 and −2 SD, a weight loss of 5–10% from baseline, or a MUAC between the 10th and 25th percentile for age and sex [9,10].
- Moderate malnutrition is defined as a BMI-for-age z-score between −2 and −3 SD, a weight loss of 10–20% from baseline, or a MUAC between the 3rd and 10th percentile [9,10].
- Severe malnutrition is defined as a BMI-for-age z-score below −3 SD, a weight loss exceeding 20% from baseline, or a MUAC below the 3rd percentile [9,10].

In pediatric oncology patients, severity classification should be interpreted in the context of the underlying disease and treatment trajectory, as tumor bulk, fluid shifts, and corticosteroid-induced weight changes may mask true nutritional depletion. Body composition assessment, particularly lean mass evaluation, is therefore recommended alongside anthropometric indices when available [7,11].

Overall, nutritional evaluation is relatively straightforward and can be tailored to the resources available within a given institution, particularly since clinical and laboratory evaluations are routinely performed by the pediatric oncologist as part of the diagnostic work-up and therapeutic management.

Incorporating structured nutritional evaluation into standard care gives pediatric oncology teams the capacity to implement appropriate nutritional interventions at the right time. A proactive approach to nutritional management appears to confer additional benefits across all age groups with malignant disease, especially considering that alterations in nutritional status may influence both the pharmacokinetics and pharmacodynamics of anticancer agents [11].

**Figure 1:**
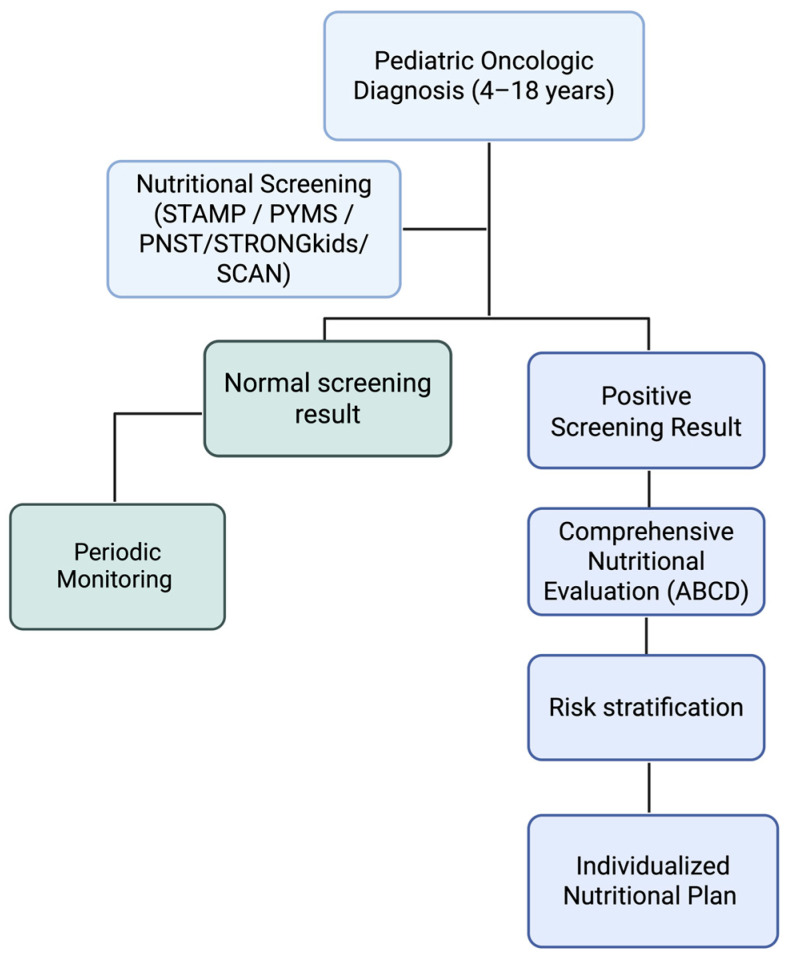
Nutritional screening and assessment of nutritional status: clinical implementation algorithm (flowchart). Created in BioRender (Avramescu, I. (2026), https://BioRender.com/f27yotm accessed on 5 June 2026).

Nutritional screening allows early identification of pediatric oncology patients at risk of malnutrition and facilitates timely nutritional intervention. Because nutritional status may change during treatment due to disease progression and therapy-related toxicities, screening should be repeated at each hospital admission and periodically during treatment and follow-up. Validated pediatric screening tools such as the Screening Tool for the Assessment of Malnutrition in Paediatrics (STAMP), the Paediatric Yorkhill Malnutrition Score (PYMS), the Paediatric Nutritional Screening Tool (PNST), the Screening Tool for Risk of Nutritional Status and Growth (STRONGkids) and the Nutrition Screening Tool for Childhood Cancer (SCAN) can support systematic risk identification, with screening frequency adapted to the patient’s clinical condition and individual risk factors [12,13,14]. The clinical implementation algorithm for nutritional screening and assessment is presented in Figure 1.

Regardless of the instrument used, nutritional risk stratification should consider both underlying disease and the anticipated treatments and therapies, as each may independently contribute to the risk of undernutrition or overnutrition [2].

### 3.2. Energy and Protein Requirements

Direct measurement of resting energy expenditure using indirect calorimetry represents the most accurate method for determining energy requirements; however, this technique is not routinely available in many clinical settings. In such cases, total energy expenditure can be estimated using reference values derived from healthy children of similar age, sex, and body composition. These estimates should be interpreted cautiously in pediatric oncology patients, as metabolic demands may vary depending on disease status, treatment intensity, inflammation, and nutritional risk [15].

Based on data synthesized from FAO/WHO reference tables, the following approximate daily caloric intake values per kilogram of body weight may be considered (Table 1).

The European Food Safety Authority (EFSA) emphasizes that energy requirements should be determined according to age, sex, body weight and/or height, and level of physical activity (PAL), while regular monitoring of body weight and growth charts remains essential [16].

In practical terms, estimated energy requirements may be adjusted by a factor of ×0.85 in sedentary or hospitalized children and by a factor of ×1.15 in those with increased physical activity levels or who engage in regular sports.

In general, the recommended daily energy intake for healthy preschool- and school-aged children is as follows (Table 2) [17]:

In healthy adolescents, resting energy expenditure is estimated to be approximately 1.400 kcal/day in females and 1.600 kcal/day in males. Total energy requirements increase to approximately 2.300–2.700 kcal/day, depending on the level of physical activity [18].

Adequate protein intake is essential in pediatric oncology patients to support growth, maintain lean body mass, and promote tissue repair during intensive anticancer therapies. In most cases, protein requirements are comparable to those of healthy children. In stable patients on standard therapy, a daily intake of 1.0–1.5 g/kg/day is sufficient, with frequent monitoring to prevent treatment-related deficits [2,19]. However, higher intake may be necessary in situations of increased metabolic stress, inflammation, or hypercatabolic states associated with treatment, including critical illness, HSCT, significant malnutrition—the minimum recommended intake is 1.5 g/kg/day, with progressive escalation up to 2.5 g/kg/day [20,21,22]. Adjustments should therefore be individualized according to nutritional status, treatment phase, and overall clinical condition, while renal function should be considered when determining protein intake [15].

Based on data synthesized from FAO/WHO, the following daily protein intake values per kilogram of body weight may be considered for healthy children (Table 3):

In general, the recommended daily protein intake for healthy children of preschool and school age is as follows (Table 4) [17]:

In healthy adolescents, daily protein requirements are primarily driven by anabolic demands associated with growth. As a rule, a daily protein intake of approximately 0.9 g/kg body weight is recommended for boys (corresponding to an average intake of about 60 g/day) and 0.8 g/kg body weight for girls (corresponding to an average of approximately 45 g/day) [23].

Although the optimal distribution of energy derived from carbohydrates and fats should generally align with that recommended for healthy children of the same age and BMI, adjustments may be considered in pediatric oncology patients at nutritional risk who present with insulin resistance or metabolic syndrome, including treatment-induced forms. In such cases, increasing the proportion of energy derived from fats relative to carbohydrates may be appropriate to enhance dietary energy density and reduce glycemic load. Tumor burden and systemic inflammation are recognized determinants of altered energy metabolism in children with cancer. Elevated levels of pro-inflammatory cytokines, including interleukin-6, tumor necrosis factor-alpha, and *C*-reactive protein, are associated with increased resting energy expenditure, accelerated protein catabolism, and impaired anabolic response to nutritional support [11,15]. Clinicians should therefore interpret standard reference values with caution in patients with active systemic inflammation and consider upward adjustment of energy and protein targets in proportion to the degree of inflammatory burden, as reflected by clinical assessment and available biochemical markers. Treatment modality represents an additional modifier of nutritional requirements. Intensive chemotherapy regimens, particularly those involving high-dose alkylating agents, anthracyclines, or prolonged corticosteroid exposure, are associated with increased protein catabolism, gastrointestinal mucosal damage, and altered substrate utilization [15,21]. Radiotherapy to the abdomen, pelvis, or gastrointestinal tract may impair nutrient absorption and increase metabolic stress, particularly during and immediately after treatment [15]. The available evidence does not currently support the application of specific numerical adjustment factors for tumor burden, inflammatory markers, or individual treatment modalities in children, and providing such values without an adequate evidence base could be clinically misleading. Individualized assessment, combining indirect calorimetry where available, serial monitoring of weight and body composition, and clinical judgment, remains the most appropriate approach to refining nutritional targets in this population. Derivation of validated adjustment coefficients for pediatric oncology patients represents an important priority for future research [11,15].

### 3.3. Vitamins and Minerals

Micronutrient intake in pediatric oncology patients should generally be aligned with established dietary reference values to support normal metabolic and physiological functions. Routine use of high-dose vitamin or mineral supplementation is not usually indicated unless a specific deficiency or clinical indication has been identified. Targeted supplementation may be required in selected cases based on laboratory findings, treatment-related factors, or individual nutritional risk. Vitamin D deficiency is relatively common in children with cancer and may require particular attention due to its role in bone health and potential impact on long-term outcomes [2,15].

In general, the recommended daily micronutrient intake for healthy preschool and school-aged children and adolescents is as follows (Table 5) [17]:

Micronutrient abnormalities are frequently reported in children with cancer and may adversely affect treatment tolerance and clinical outcomes. The most consistently documented deficiencies include vitamin D, folate, selenium, zinc, and ferritin or iron-related markers [24,25,26,27,28]. In patients with food insecurity, restricted diets, or poor dietary diversity, vitamin A, vitamin B12, folate, and iron should also be considered as priorities. Routine comprehensive micronutrient testing may not be feasible in all institutional settings due to resource and funding constraints. Prioritized assessment and targeted supplementation are therefore recommended, with laboratory monitoring performed when clinically indicated and when local resources allow. While general micronutrient reference intakes provide a baseline for nutritional planning, specific treatments in pediatric oncology are associated with increased risk of targeted deficiencies that warrant clinical vigilance and, where indicated, prophylactic or therapeutic supplementation. Methotrexate-based chemotherapy regimens directly impair folate metabolism through dihydrofolate reductase inhibition. Patients receiving high-dose methotrexate require leucovorin rescue, and folate status should be monitored throughout treatment. Supplementation with folic acid should be guided by clinical protocol and laboratory findings, as unsupervised supplementation may interfere with methotrexate efficacy [5]. Intensive chemotherapy and radiotherapy, particularly regimens involving platinum-based agents, ifosfamide, or abdominal and pelvic irradiation, are associated with significant urinary and gastrointestinal losses of zinc and magnesium, as well as impaired calcium absorption. Zinc deficiency may manifest as impaired wound healing, immune dysfunction, and dysgeusia, further compromising nutritional intake. Calcium and magnesium levels should be monitored, when clinically indicated and resources allow, in patients receiving nephrotoxic agents or abdominal radiotherapy, with supplementation initiated based on documented deficiency or significant ongoing losses [5,15]. Bone health deserves particular attention in HSCT recipients, who are at elevated risk of osteopenia and osteoporosis due to the combined effects of conditioning regimens, prolonged corticosteroid use for graft-versus-host disease prophylaxis and treatment, reduced physical activity, and vitamin D deficiency. Baseline and periodic assessment of 25-hydroxyvitamin D levels, calcium, phosphate, and alkaline phosphatase is recommended in this population, with supplementation adjusted accordingly. Dual-energy X-ray absorptiometry should be considered for longitudinal bone density monitoring in patients receiving prolonged immunosuppressive therapy [5,15,21]. Beyond these specific contexts, clinicians should maintain awareness of the broad micronutrient depletion that can accompany prolonged reduced oral intake, mucositis, diarrhea, and malabsorption across all treatment phases. Laboratory monitoring should be prioritized for patients at highest clinical risk, including those undergoing HSCT, receiving high-dose methotrexate, prolonged corticosteroid therapy, or nephrotoxic agents such as platinum compounds or ifosfamide; those receiving abdominal or pelvic radiotherapy; and those presenting with persistent mucositis, diarrhea, malabsorption, prolonged reduced oral intake, severe malnutrition, or clinical signs of deficiency. Where comprehensive biochemical testing is not available, clinical assessment and dietary evaluation should guide supplementation decisions [15,21].

### 3.4. Physical Activity

Regular physical activity is safe for pediatric oncology patients and is associated with improved functional capacity, muscle mass, bone health, quality of life, and psychological well-being. Exercise prescriptions should be individualized according to age, clinical condition, and hematological parameters. Programs should be supervised and adapted to the patient’s age, disease stage, treatment modality, and temporary contraindications such as severe thrombocytopenia, febrile neutropenia, uncontrolled pain, or active infections. Physical activity interventions should ideally be initiated within hospital-based physiotherapy programs and supervised by a trained physiotherapist or continued in structured medical rehabilitation settings.

Adolescents (12–18 years) may generally follow exercise prescriptions similar to those recommended for adults (moderate-to-vigorous intensity, including both aerobic and resistance training). In school-aged children, activity should be adapted (e.g., active play and moderate-intensity exercises), while in preschool-aged children the focus should be on maintaining daily movement and play rather than structured training sessions [15,20,29]. In adolescents, who may be managed according to adult-oriented physical activity principles, the promotion of both aerobic and resistance exercise results in significantly greater improvements in upper and lower body muscle strength compared with usual care. Emerging evidence also suggests that resistance training may be more effective than aerobic exercise alone in enhancing muscle strength [15].

### 3.5. Nutritional Interventions

**Figure 2:**
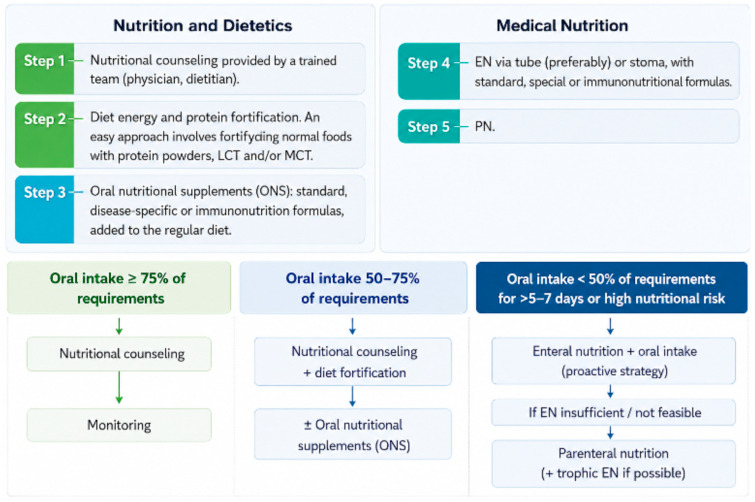
Escalation of nutritional intervention in pediatric oncology patients at nutritional risk: Implementation algorithms. Created in BioRender (Avramescu, I. (2026), https://BioRender.com/jso1ae5 accessed on 14 May 2026).

The primary objectives of nutritional intervention in pediatric oncology are to support and promote adequate growth and development during anticancer treatment, to maintain optimal nutritional status, to correct existing nutritional abnormalities, and to prevent future disturbances that may arise during intensive multimodal therapy [30]. The nutritional intervention escalation algorithm is presented in Figure 2.

Although nutritional strategies aimed at increasing energy and protein intake are required in cases of insufficient dietary intake, it is important to recognize that special “anticancer diets” are commonly adopted by families of oncology patients [20]. When such diets involve energy restriction, they should be actively discouraged. Ketogenic diets—and especially calorie-restricted regimens—should be avoided, as their efficacy remains insufficiently validated and they may represent potentially harmful strategies that could negatively interfere with the effectiveness of active oncologic therapies in patients at risk of malnutrition or sarcopenia, as well as adversely affect long-term outcomes in childhood cancer survivors [31].

Fasting and caloric restriction in the peri-treatment setting are not currently supported by robust evidence-based clinical data [32].

If a patient is adequately nourished, does not experience weight loss, and consumes at least 50% of the recommended nutritional intake, nutritional counseling provided by a pediatric gastroenterologist or a dietitian trained in oncology nutrition may be sufficient.

Oral nutritional intervention should include both symptom management strategies (addressing conditions that impair intake) and dietary guidance, including energy and protein fortification and/or the use of ONS. The addition of long-chain fatty acids or medium-chain triglycerides (MCT), as well as protein powders, to preferred foods may represent practical options. Nutrient-dense traditional foods and home-prepared supplements may also be effective strategies to overcome economic or cultural barriers [2].

ONS should be introduced in pediatric oncology patients who are unable to meet their nutritional requirements through normal oral intake, even if fortified. If the patient is unable to achieve at least 50% of daily requirements through regular oral intake, ONS should be initiated directly without relying only on dietary fortification [2].

Most ONS are available as ready-to-use liquid formulas (less commonly as powders), provide complete macro- and micronutrient profiles and may be used either as supplements to insufficient intake or as a sole source of nutrition. They typically provide 1–2 kcal/mL, are available in various flavors (including neutral options) and may be incorporated into preferred meals [19,30,33].

Formulas may be:

- Standard/polymeric (containing intact proteins and long-chain fatty acids) for patients with functional gastrointestinal tracts.
- Special/oligomeric (containing amino acids or oligopeptides and MCT) for patients with malabsorption or maldigestion.
- Immunonutrition-enriched, containing pharmaconutrients such as arginine, omega-3 fatty acids, nucleotides, or antioxidants.
- Modified-viscosity formulations for patients with oropharyngeal dysphagia.

Long-chain omega-3 fatty acids have been associated with improvements in appetite, body weight, postoperative morbidity, and quality of life. They may help stabilize or improve appetite, increase dietary intake, enhance lean body mass, and support overall weight maintenance [15,21].

When multiple enteral formula options are clinically appropriate, selection should follow a stepwise priority framework. Standard/polymeric formulas are the first choice in patients with an intact and functional gastrointestinal tract. Special/oligomeric formulas should be reserved for patients with documented malabsorption, severe mucositis, short bowel syndrome, or persistent diarrhea [15,34].

In pediatric oncology patients at high nutritional risk who are still able to eat, early initiation of enteral nutrition together with oral intake may be appropriate if treatment-related adverse effects or disease-related conditions are expected to compromise oral intake. When enteral nutrition (EN) is difficult to implement, complementary parenteral nutrition (PN) may be considered.

Enteral nutrition should generally be considered in the following situations:

- When oral intake remains below 50% of estimated requirements for more than 5 consecutive days. However, a proactive strategy is preferred in high-risk patients [22]. EN should be initiated if the patient is expected to be unable to eat for more than 7 days or if intake below 60% of estimated energy expenditure is anticipated for more than 10 days [21]. EN should compensate for the gap between actual intake and calculated needs.
- When the child is already malnourished.
- When there is >5% weight loss since diagnosis.
- When the child crosses two growth percentiles during treatment [35,36].

Maintaining gastrointestinal function and metabolic homeostasis during active anticancer therapy remains a major challenge in pediatric oncology [30]. Continuous enteral feeding should be initiated at 1–2 mL/kg/hour in infants and younger children, and at 20–25 mL/hour in older children and adolescents, not exceeding 25% of the calculated daily target on the first day. The infusion rate should be advanced by 10–25 mL/hour every 8–12 h as tolerated, with the goal of reaching full nutritional targets within 48–72 h. In patients with compromised gastrointestinal function or following prolonged periods of reduced intake, a more gradual progression over 3–5 days is recommended to minimize the risk of refeeding complications and gastrointestinal intolerance. Once full enteral tolerance is established, transition from continuous to bolus feeding may be attempted, beginning with 4–6 boluses per day and adjusting volume and frequency according to patient tolerance and daily schedule [34,37]. Diarrhea is the most common complication of EN in pediatric oncology patients and may result from formula composition, infusion rate, antibiotic exposure, or underlying gastrointestinal toxicity. Initial management should include reduction of infusion rate by 25–50%, verification of formula temperature and administration technique, and exclusion of infectious causes. If diarrhea persists, transition to a semi-elemental or monomeric formula with reduced osmolarity and higher MCT content should be considered. Probiotics may be used cautiously in selected patients, with awareness of the immunosuppression-related risks in this population [34,37]. Abdominal distension and delayed gastric emptying are frequently encountered during enteral feeding, particularly in patients receiving opioids, undergoing chemotherapy, or following abdominal surgery. Management should include temporary reduction of infusion rate, elevation of the head of the bed to 30–45 degrees, assessment of gastric residual volume where clinically indicated, and consideration of prokinetic agents. If intragastric feeding remains poorly tolerated despite these measures, post-pyloric feeding via nasojejunal tube should be considered. PN should be initiated or supplemented if enteral targets cannot be achieved within 5–7 days despite route and formula optimization [34].

Combined EN and PN may be particularly useful in:

- Radiotherapy-associated malabsorption, diarrhea, xerostomia, mucositis, dysphagia, ileus, intestinal inflammation, or bleeding.
- Surgical interventions leading to motility disorders, short bowel syndrome, enzymatic insufficiency, digestive failure, or metabolic dysregulation.
- Chemotherapy-induced malabsorption, diarrhea, nausea, vomiting, anorexia, mucositis, gastroenteritis, or metabolic disturbances.
- Broad-spectrum antibiotic therapy associated with significant microbiome disruption and severe antibiotic-associated diarrhea.

Enteral nutrition via nasogastric or percutaneous access is recommended in cases of anticipated severe mucositis and in tumors of the head, neck, or thorax that obstruct food passage or interfere with swallowing.

Dysphagia should be identified and treated early. In cases of prophylactic EN, patients should be encouraged and educated to maintain swallowing reflex function. Jejunal enteral access may be considered when intragastric feeding is contraindicated [2].

Enteral formulas are generally similar or identical to those used as ONS. Although home enteral nutrition using blended real foods may be feasible if strict safety and compositional standards are met, ESPGHAN recommends the use of commercially prepared enteral formulas. These provide sterile, well-defined macro- and micronutrient compositions, adjusted to the patient’s needs, in a low-viscosity liquid form suitable for tube administration, and are associated with lower complication rates [30,34].

**Figure 3:**
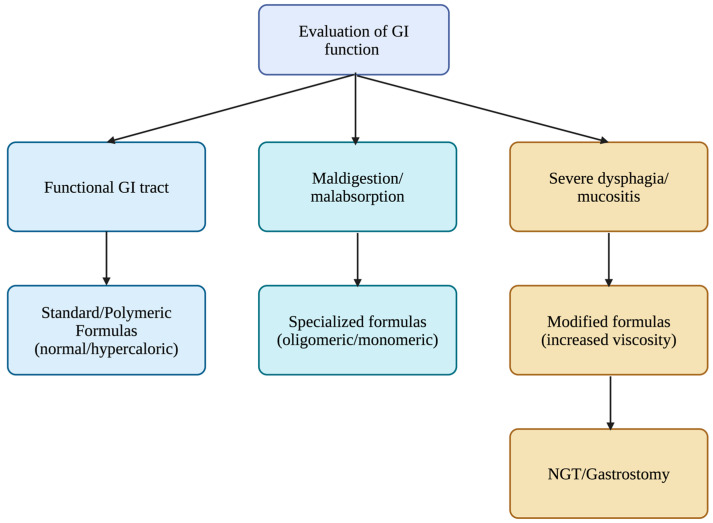
Selection of the route and formula for enteral nutrition. Created in BioRender (Avramescu, I. (2026), https://BioRender.com/szrj42n accessed on 14 May 2026).

In patients with a functional gastrointestinal tract, standard polymeric formulas (intact proteins and long-chain fatty acids), either normocaloric or hypercaloric, are appropriate. In patients with malabsorption, specialized oligomeric or monomeric formulas (containing oligopeptides or amino acids and medium-chain triglycerides) should be selected, also in normocaloric or hypercaloric variants. The route and formula selection algorithm is presented in Figure 3.

The parenteral route is indicated when enteral nutrition is not feasible or remains inadequate for more than 5–7 days. During parenteral nutrition, minimal enteral feeding should be reintroduced as early as possible to promote gastrointestinal integrity and support a healthy microbiome. Careful monitoring for metabolic and infectious complications is mandatory.

Refeeding syndrome represents a potentially life-threatening metabolic complication triggered by the reintroduction of nutrients following a prolonged period of starvation or severely restricted caloric intake. Refeeding syndrome remains underdiagnosed due to variability in diagnostic criteria, overlap with other treatment-related complications, and limited awareness [38]. In the pediatric oncology population, where extended inadequate intake, disease-related metabolic stress, and intensive anticancer treatment combine, the risk of refeeding syndrome is considerable, and prevention is essential.

All patients should be screened for refeeding syndrome risk prior to nutritional support initiation. The most important risk factors include low BMI-for-age, significant weight loss, prolonged inadequate intake of five or more days, low baseline electrolyte levels, and prior chemotherapy exposure [30,34].

**Figure 4:**
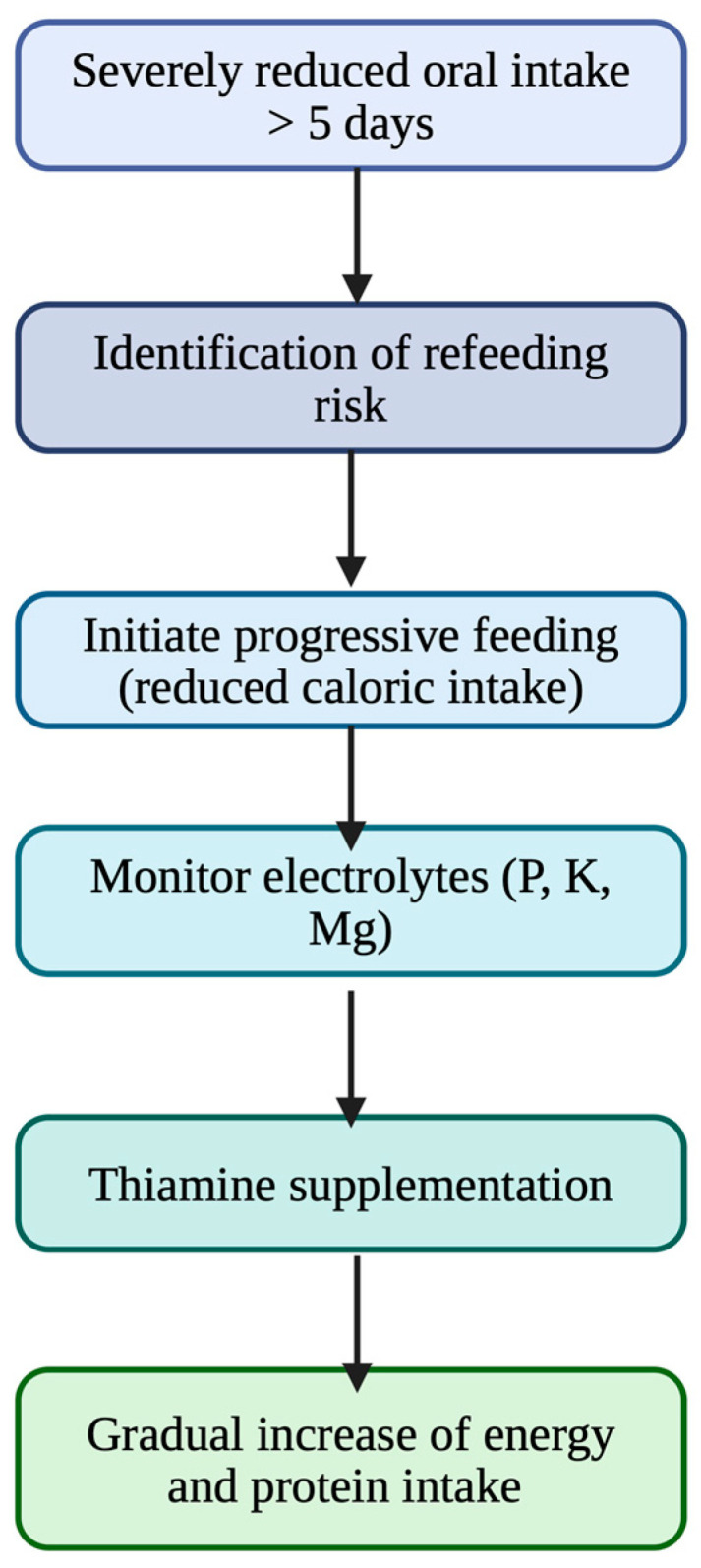
Prevention of refeeding syndrome. Created in BioRender (Avramescu, I. (2026), https://BioRender.com/m6j9w9j accessed on 14 May 2026).

Nutritional reintroduction should begin at 40–50% of calculated caloric goals, with an initial glucose infusion rate of 4–6 mg/kg/min, increased by 1–2 mg/kg/min daily to reach the maximum target: 14–18 mg/kg/min over 5–10 days [37]. A serum phosphate level of ≤0.6 mmol/L or a reduction exceeding 30% within 72 h of refeeding initiation suggests imminent refeeding syndrome and requires immediate caloric reduction. Electrolyte monitoring should be performed before feeding initiation and every 12 h during the first three days, with prophylactic supplementation of potassium (2–4 mmol/kg/day), phosphate (0.3–0.6 mmol/kg/day), and magnesium (0.2–0.4 mmol/kg/day) [38]. Thiamine should be administered at 2 mg/kg/day (maximum 100–200 mg/day) before refeeding begins and continued for 5–7 days [37]. Vital signs should be monitored every four hours during the first 24 h, with daily weight measurement, strict fluid balance recording, and cardiorespiratory monitoring in severe cases. EN is preferred whenever gastrointestinal function is preserved. The same refeeding principles apply regardless of the nutritional route selected, as illustrated in Figure 4 [37].

In patients in whom refeeding syndrome develops, energy intake should be reduced. Both the ASPEN consensus and NICE guidelines recommend a careful approach to nutrient reintroduction, with ASPEN recommending an initial intake of 10–20 kcal/kg on the first day, and NICE proposing an even more cautious strategy of no more than 10 kcal/kg/day, or 5 kcal/kg/day in patients at particularly high risk [37,39].

### 3.6. Special Situations

#### 3.6.1. Nutritional Interventions During Active Anticancer Treatment

A balanced nutritional approach, aligned with age-specific energy and protein requirements and adapted to clinical status, together with encouragement of daily physical activity adjusted to individual capacity, contributes to the preservation of muscle mass, reduction of fatigue, and optimization of response to anticancer therapy.

In palliative settings, nutritional support should be individualized, with primary goals focused on comfort, adequate hydration, and maintenance of quality of life.

#### 3.6.2. Nutritional Interventions During Intensive Chemotherapy for High-Risk Cancers and Hematopoietic Stem Cell Transplantation

**Figure 5:**
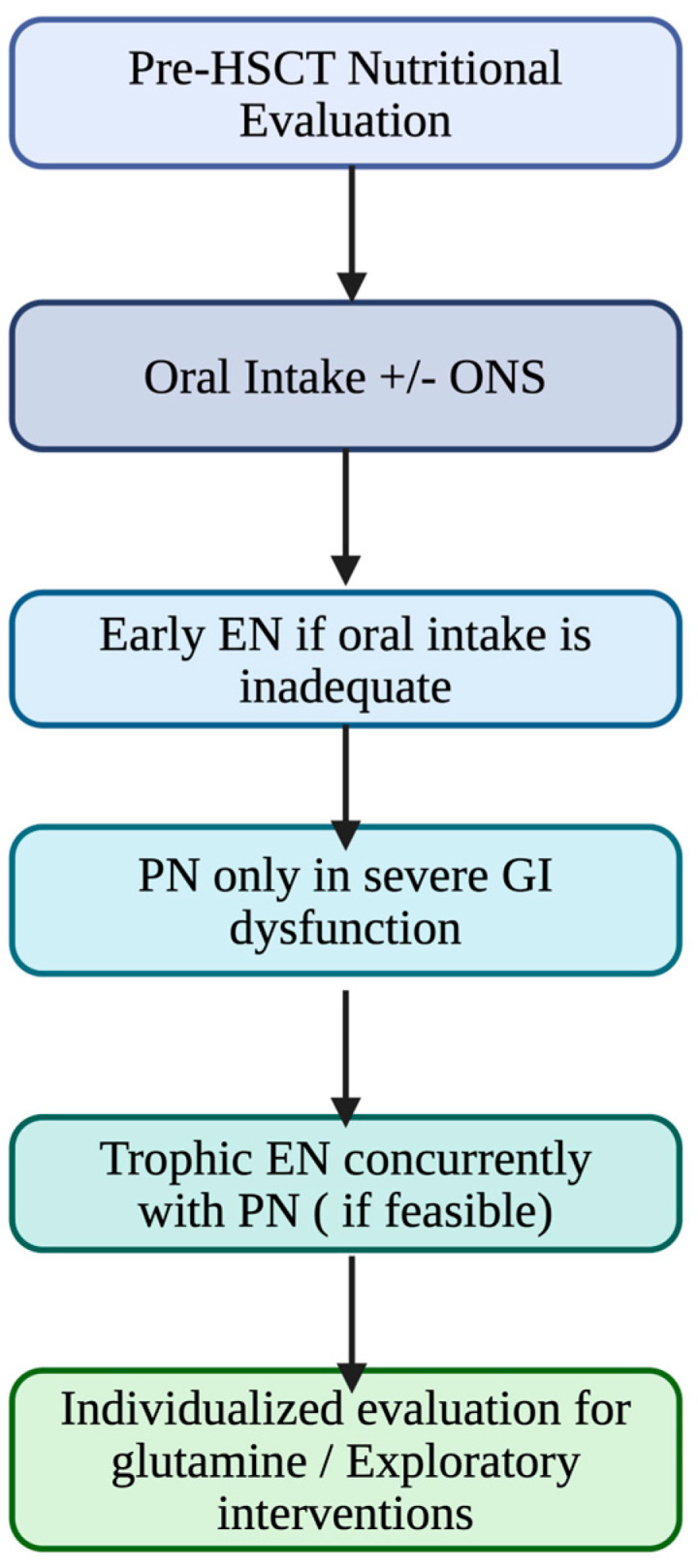
Nutritional support in HSCT. Created in BioRender (Avramescu, I. (2026), https://BioRender.com/wx3lwbj accessed on 14 May 2026).

Parenteral nutrition should be initiated only in cases of severe gastrointestinal dysfunction (e.g., severe mucositis, intestinal obstruction or ileus, severe malabsorption, prolonged diarrhea, or symptomatic gastrointestinal graft-versus-host disease) and should be limited to the shortest possible duration. If not contraindicated, at least minimal trophic enteral feeding should be maintained concurrently with parenteral nutrition. The nutritional management approach in patients undergoing HSCT is summarized in Figure 5.

Glutamine supplementation may be considered in selected HSCT recipients receiving PN, particularly when severe mucositis limits oral intake. The available evidence suggests a possible reduction in mucositis-related supportive care needs, including a shorter duration of total PN in pediatric HSCT [40]. Pharmacologic doses of glutamine may provide potential benefits in preserving intestinal mucosal integrity and reducing the severity of mucositis.

Butyrate supplementation may be considered in patients undergoing allogeneic HSCT. In accordance with recent evidence in the literature and supported by our current clinical experience, postbiotic nutritional intervention consisting of butyrate supplementation at 800–1200 mg/day may reduce the incidence of GVHD in pediatric oncology patients undergoing allogeneic HSCT. Similar findings have recently been reported by Kim et al. (Transplantation and Cellular Therapy, 2025), who demonstrated a trend toward reduced cumulative incidence of all-grade GVHD in the treated group compared to controls, with the authors suggesting that butyrate supplementation may have a preventive role in acute GVHD [41].

Ensuring optimal nutrition in pediatric oncology patients is critical for their overall care and recovery. While low-bacterial-load diets are often suggested in specific clinical scenarios, it is imperative to prioritize strict adherence to food hygiene standards. Except for pediatric oncology patients within the first 30 days following allo-HSCT, low-bacterial-load diets have not demonstrated additional benefit compared with strict compliance with food hygiene measures and safe food preparation practices.

#### 3.6.3. Nutritional Interventions During Radiotherapy

Routine use of enteral and/or parenteral nutritional support is not recommended in all patients undergoing radiotherapy [21]. In cases involving irradiation of the head and neck, thorax, or gastrointestinal tract, special attention should be given to achieving adequate nutritional intake [15]. All patients at risk of dysphagia should be assessed before initiation of radiotherapy and monitored regularly during treatment and follow-up [15]. Nutritional management may include texture-adapted diets, higher-viscosity oral nutritional solutions, enteral nutrition, and/or parenteral nutrition. Food consistency and viscosity should be tailored to the severity of dysphagia.

Parenteral nutrition is indicated if oral or enteral tolerance is insufficient. All patients with dysphagia who are not receiving oral feeding should perform supervised swallowing exercises under guidance [15].

#### 3.6.4. Nutritional Interventions in Pediatric Oncologic Surgery

Specialized perioperative nutritional support reduces infectious complications and improves postoperative recovery in malnourished pediatric oncology patients. A short period of preoperative nutritional optimization (approximately 7–14 days) may be beneficial when clinically feasible [2,15].

Common pharmaconutrients included in immunonutrition formulas are arginine, omega-3 fatty acids, and nucleotides/ribonucleotides. Although one meta-analysis demonstrated a significant reduction in infectious complications with exclusive preoperative immunonutrition compared with a normal diet or standard ONS [42], clear evidence supporting exclusive use of immunonutrition over standard oral supplements in the preoperative setting remains limited [43].

#### 3.6.5. Nutrition in Cancer Survivors and Young Adults

Survivors at nutritional risk may benefit from monthly evaluation; overweight and obese survivors from quarterly evaluation; and those without risk factors from evaluation every six months during the first year of follow-up and annually thereafter. Childhood cancer survivors are at increased risk of obesity, dyslipidemia, hyperglycemia, metabolic syndrome, osteopenia, and osteoporosis [2]. Coronary heart disease, stroke, and heart failure are among the leading causes of morbidity and mortality in this population. This increased risk results from combined treatment effects and traditional cardiovascular risk factors (hypertension, diabetes, dyslipidemia, obesity) [21].

Even well-nourished survivors with metabolic risk factors (e.g., dyslipidemia, hypertriglyceridemia, unhealthy dietary habits) should undergo quarterly evaluation during the first year, biannual evaluation until year five, and annual assessment thereafter [11].

Survivors frequently exhibit sedentary behavior, fatigue, reduced physical performance, decreased muscle mass and strength, impaired mobility, and psychological stress [21]. Physical activity represents an effective strategy for improving aerobic capacity, physical fitness, and functional status [15]. Moreover, regular exercise may improve insulin resistance and glycemic control.

Dietary patterns in children with cancer often include high-calorie foods with low fiber and micronutrient content, limited diversity, and significant taste-related modifications. During treatment, permissive parenting styles may lead to sedentary behavior and unhealthy eating habits. After treatment, reversing these behaviors can be challenging, fostering a long-term obesogenic environment that may persist into young adulthood [21].

High consumption of red meat (beef, pork, lamb) has been associated with increased breast cancer risk and overall cancer mortality. Although the effect of plant-based diets on recurrence remains unclear, fruit and vegetable intake appears to provide protective effects against certain cancers. Therefore, a diet rich in fruits and vegetables should be recommended to cancer survivors [15].

Evidence from the local oncological context confirms that sedentary behavior and suboptimal dietary patterns are highly prevalent among cancer patients in Romania, reinforcing the importance of early and sustained lifestyle interventions [44].

Treatment exposure, alone or combined with adverse lifestyle factors, contributes to increased risk of obesity and cardiovascular disease among childhood cancer survivors [21]. Lifestyle interventions—including dietary improvement and promotion of physical activity—represent key opportunities to reduce chronic disease burden. The optimal timing for intervention is before the end of treatment [45].

#### 3.6.6. Palliative Care

Patients at nutritional risk in this category should be evaluated for treatable symptoms affecting nutritional status and for metabolic disturbances, as some may have life expectancy measured in months or years. Poor nutritional status may worsen performance status, quality of life, survival duration, and overall disease burden [15].

If survival is expected to extend over months or years, nutritional therapy should ensure adequate energy and protein intake, mitigate metabolic disturbances, and maintain functional performance and acceptable quality of life. In such patients who are unable to eat, medical nutrition may improve survival [46].

If expected survival is limited to weeks, interventions should be non-invasive and primarily focus on psychosocial and existential support [47]. In cases of insufficient oral intake, enteral nutrition may minimize weight loss and can be continued as long as the patient consents and has not entered the dying phase. Long-term parenteral nutrition should be initiated only when intestinal absorption is severely impaired, enteral nutrition is insufficient, expected survival exceeds three to four months, quality of life is likely to improve, and the patient explicitly requests it [21].

Care should prioritize comfort. Parenteral hydration and nutrition are unlikely to provide substantial benefit in most patients during this phase [21]. Family members may request medical nutrition or hydration; however, during terminal hypometabolism, standard energy and substrate provision may be excessive and potentially contribute to metabolic distress [48].

### 3.7. Recommendations

Recommendation 1

We recommend the systematic integration of clinical nutrition into the standard management of pediatric oncology patients.

Comprehensive nutritional evaluation and individualized nutritional support should be initiated at the time of diagnosis and maintained throughout the entire course of treatment, as well as during survivorship.

Recommendation 2

We recommend the involvement of a multidisciplinary team with expertise in pediatric oncology nutrition—such as a pediatrician and/or a pediatric gastroenterologist together with a registered dietitian—for nutritional screening, as well as for comprehensive evaluation and ongoing monitoring of nutritional status.

Recommendation 3

We recommend that nutritional screening be performed in all pediatric oncology patients at the time of diagnosis. A comprehensive nutritional evaluation should be conducted in those with a positive screening result or in the presence of major nutritional risk factors.

Nutritional screening and detailed assessment represent complementary steps. Screening enables the rapid identification of patients at risk of malnutrition, whereas comprehensive assessment provides an integrated evaluation of body composition, dietary intake, metabolic profile, and clinical status.

Recommendation 4

We recommend performing nutritional screening at every hospital admission and at regular intervals throughout treatment and post-therapeutic follow-up.

Validated screening tools such as STAMP, PYMS, PNST, STRONGkids or SCAN may be used, with adjustment of screening frequency according to clinical stability and individual risk factors.

Recommendation 5

If resting energy expenditure is not individually measured, we recommend estimating total energy requirements as comparable to those of healthy children of the same age, sex, and BMI, with individualized adjustment for patients at nutritional risk.

Recommendation 6

We suggest that, in the absence of renal insufficiency, protein intake in pediatric oncology patients should be similar to that of healthy children of the same age. Based on the results of nutritional assessment and the individual clinical and therapeutic context, protein intake may be increased up to 2.5 g/kg/day.

Recommendation 7

We recommend maintaining a balanced macronutrient distribution, with the possibility of increasing lipid intake and reducing carbohydrate intake in patients with insulin resistance who are experiencing weight loss.

Recommendation 8

We suggest that vitamins and minerals be provided in amounts consistent with established reference nutrient intakes. Targeted supplementation is recommended in cases of documented deficiency or specific clinical indications, while routine high-dose supplementation in the absence of deficiency is not recommended. Vitamin D warrants particular attention given the high prevalence of deficiency in this population. When clinically indicated and resources allow, targeted laboratory monitoring should prioritize vitamin D, folate, selenium, zinc, and ferritin or iron status.

Recommendation 9

In the absence of an absolute temporary contraindication (e.g., platelet count < 20,000/µL), we recommend promoting daily physical activity tailored to the age and clinical status of the pediatric oncology patient.

Recommendation 10

We recommend incorporating supervised, age-appropriate resistance training in adolescents.

Recommendation 11

We suggest avoiding energy-restricted diets in pediatric oncology patients at nutritional risk.

Recommendation 12

We recommend oral nutritional intervention as the first-line approach in patients who are able to eat, including nutritional counseling, dietary fortification, and the use of oral nutritional supplements (ONS).

Recommendation 13

We recommend supplementation with long-chain omega-3 fatty acids or fish oil in patients presenting with progressive, unintentional weight loss.

Recommendation 14

We recommend a proactive enteral nutrition strategy in patients at high nutritional risk when reduced oral intake is anticipated.

Recommendation 15

We recommend nasogastric tube feeding as the first-line option for enteral nutrition, with consideration of gastrostomy for prolonged support.

Recommendation 16

We recommend initiating enteral nutrition using continuous administration, with subsequent transition to bolus feeding if well tolerated.

Recommendation 17

We recommend selecting the enteral formula according to gastrointestinal functionality and patient age.

Recommendation 18

We recommend parenteral nutrition (PN) as a rescue intervention when enteral nutrition (EN) is impossible or insufficient.

Recommendation 19

We recommend the gradual escalation of nutritional intake in patients with severely reduced intake in order to prevent refeeding syndrome.

Recommendation 20

We recommend maintaining adequate nutritional intake and age-appropriate physical activity throughout anticancer treatment.

Recommendation 21

We recommend escalation of nutritional support from oral to enteral and parenteral routes according to tolerance and clinical requirements.

Recommendation 22

We recommend intensive nutritional monitoring in patients undergoing intensive chemotherapy and hematopoietic stem cell transplantation (HSCT).

Recommendation 23

We recommend prioritizing enteral nutrition over parenteral nutrition whenever the gastrointestinal tract is functional.

Recommendation 24

Glutamine supplementation may be considered in patients undergoing HSCT who require parenteral nutrition.

Recommendation 25

We do not recommend the routine use of low-bacterial-load (neutropenic) diets; instead, we recommend strict adherence to food hygiene and safe food-handling practices.

Recommendation 26

We recommend maintaining adequate nutritional intake during radiotherapy in order to prevent deterioration of nutritional status and avoid treatment interruptions.

Recommendation 27

We suggest early identification and prompt management of dysphagia.

Recommendation 28

We suggest prioritizing enteral nutrition over parenteral nutrition in cases of severe radiation-induced mucositis and in obstructive tumors of the thorax, head, and neck.

Recommendation 29

There is insufficient high-quality evidence to recommend glutamine supplementation for the prevention of radiation-induced enteritis, diarrhea, stomatitis, esophagitis, or skin toxicity, nor to support the routine use of probiotics for the reduction of diarrhea.

Recommendation 30

We suggest specialized nutritional support before surgery, during hospitalization, and after discharge in all surgical pediatric oncology patients who are malnourished or at nutritional risk.

Recommendation 31

We suggest 7–14 days of preoperative nutritional support in malnourished pediatric oncology patients undergoing major surgical procedures.

Recommendation 32

We suggest the use of oral or enteral immunonutrition formulas in malnourished pediatric oncology patients undergoing major oncologic surgery.

Recommendation 33

We suggest continuing nutritional screening in childhood cancer survivors, with comprehensive nutritional assessment performed in those with a positive screening result or major nutritional risk factors.

Recommendation 34

We suggest that cancer survivors engage in regular physical activity adapted to age and physical status.

Recommendation 35

We suggest that cancer survivors maintain optimal body weight and adopt a healthy lifestyle, avoiding tobacco use and following a diet rich in vegetables, fruits, whole grains, and high-quality protein from minimally processed sources, while limiting saturated fats, red meat, and alcohol.

Recommendation 36

We suggest initiating appropriate lifestyle and dietary modifications during the survivorship transition phase, before completion of oncologic treatment.

Recommendation 37

We suggest continuing nutritional screening in patients receiving palliative care, with comprehensive nutritional assessment performed in those with a positive screening result or major nutritional risk factors.

Recommendation 38

We suggest implementing nutritional interventions in advanced cancer only after shared decision-making (SDM), considering disease prognosis, expected quality-of-life benefits, survival potential, and the burden of nutritional care.

Recommendation 39

We suggest prioritizing oral nutritional supplements and enteral nutrition whenever feasible.

Recommendation 40

We suggest short-term, limited parenteral hydration in patients entering the dying phase who develop acute confusion, to exclude dehydration as a precipitating factor.

Exploratory Recommendation

Butyrate supplementation may be considered in patients undergoing allogeneic HSCT, based on emerging evidence and limited clinical experience.

This statement is based on preliminary evidence and current clinical experience only, does not represent the standard of care, and requires individualized clinical evaluation and prospective validation before broader implementation.

## 4. Discussion

Improving nutritional care for children with cancer remains one of the most important but least evidence-based domains of supportive care in pediatric oncology. The present evidence shows that nutritional status influences treatment tolerance, risk of infection, and long-term survival outcomes. There are different clinical approaches across centers regarding the frequency of nutritional screening, assessment tools, intervention thresholds, and monitoring protocols.

There is a gap in pediatric oncology supportive care caused by the absence of a clinical protocol for nutritional management applicable throughout the treatment period and survivorship. Our Delphi consensus recommendations are in line with two key reference documents in the field, while extending their scope and practical utility in several meaningful directions [2,30].

The recommendations presented in our consensus are aligned with existing international guidance in pediatric oncology nutrition, including the consensus statement by Fabozzi et al. (2022) [2] and the Delphi-derived recommendations by Budka-Chrzęszczyk et al. (*Nutrients*, 2024) [30]. These documents highlight similar principles in nutritional care in this population: the dual burden of undernutrition and overnutrition, the central role of the multidisciplinary team, the value of validated screening tools, and the importance of body composition assessment beyond weight-based metrics. Rather than duplicating these contributions, our consensus document extends them in several clinically meaningful directions: a comprehensive, integrated framework covering nine thematic domains across the full treatment trajectory; quantitative, age- and sex-stratified energy and protein reference tables; an explicit refeeding syndrome prevention and management protocol; structured guidance for survivorship and palliative care; and an exploratory recommendation on postbiotic supplementation grounded in emerging biological and clinical evidence. In addition, the present consensus makes several contributions that extend beyond the reference documents.

Using a formal Delphi process provides a higher level of methodological rigor and reproducibility. The Delphi methodology is well-suited to clinical domains where high-quality randomized evidence is limited, as it integrates expert knowledge while minimizing the influence of dominant voices and limiting response bias due to social desirability.

Our recommendations integrate both assessment and intervention management, covering the complete course from initial screening to nutritional support, with decision algorithms for each treatment modality. This includes detailed guidance for the selection of enteral formula type—polymeric versus oligomeric or monomeric, and normocaloric versus hypercaloric—based on gastrointestinal function. In addition, our document provides age- and sex-stratified energy and protein reference tables derived from FAO/WHO and EFSA sources, including practical adjustments for physical activity level. This provides practical clinical tools to reduce the decision-making burden for physicians.

Another contribution is the inclusion of a protocol for the prevention and management of refeeding syndrome, a potentially life-threatening complication that is underrecognized in pediatric oncology. Our recommendations define patients at risk, describe a gradual reintroduction of caloric and protein intake, and specify the role of electrolyte monitoring and prophylactic thiamine supplementation.

The present consensus highlights emerging evidence on postbiotic nutritional intervention. Our exploratory recommendation regarding butyrate supplementation in pediatric allogeneic HSCT recipients is supported by data published by Kim et al. in *Transplantation and Cellular Therapy* (2025) [41], reporting a trend toward reduced cumulative GVHD incidence. This recommendation is defined as exploratory, acknowledging the limited current evidence and the need for individualized clinical evaluation.

In the present document, the use of low-bacterial-load diets is limited to the first 30 days following allogeneic HSCT, rather than rejecting such diets entirely. Beyond this period, strict adherence to food hygiene measures is considered equally effective, allowing greater dietary diversity while improving quality of life. Families and caregivers should be counseled to avoid raw or undercooked meat, fish, and eggs; exclude unpasteurized dairy products and juices; thoroughly wash all fresh produce; and maintain safe food storage and preparation practices, including appropriate refrigeration and separation of raw and cooked foods.

Finally, the structured inclusion of palliative care as a distinct clinical domain addresses one of the most ethically and clinically challenging areas of pediatric oncology practice. Our document presents prognosis-stratified criteria for the initiation, maintenance, and limitation of artificial nutritional support. Nutritional decisions at end of life involve complex interactions between clinical prognosis, patient and family values, cultural expectations, and the potential for nutritional interventions to cause harm through metabolic overload in the context of terminal hypometabolism. Clear, consensus-derived guidance in this area provides clinical teams with a framework for approaching these decisions in a structured and ethically coherent manner.

Several limitations of the present consensus should be acknowledged when interpreting these recommendations. As with any expert consensus, the recommendations presented here are based on the collective judgment of the panel rather than direct evidence from randomized controlled trials. In pediatric oncology nutrition, high-quality prospective data remain limited, and many recommendations rely on evidence extrapolated from observational studies, adult populations, or broader pediatric settings. While the Delphi method offers a structured and widely accepted approach for developing guidance in areas where strong evidence is lacking, it is important to acknowledge the limitations inherent in expert-based recommendations. Therefore, each recommendation should be interpreted in consideration of the strength of the available evidence and the clinical context.

Second, the present consensus was developed on behalf of the Romanian Society of Pediatric Hematology and Oncology and the Romanian Society of Pediatric Gastroenterology, Hepatology and Nutrition, and is explicitly intended as a nationally adapted clinical framework for pediatric oncology nutrition in Romania. The recommendations were formulated in alignment with key international guidelines, including those published by ESPEN, ESPGHAN, SIOP, COG and the IIPAN consensus, and should be interpreted as a nationally adapted application of these international frameworks to the Romanian clinical context. Clinicians and institutions in other countries are encouraged to refer to the primary international frameworks cited throughout this document when applying these principles beyond the Romanian clinical context.

In addition, the implementation of micronutrient monitoring recommendations should be adapted to local laboratory availability, institutional funding, and individual patient risk profiles. Centers with limited access to comprehensive biochemical testing are encouraged to prioritize clinical and dietary assessment as the primary approach to identifying nutritional risk, with laboratory monitoring reserved for high-risk patients.

The lower age boundary of four years reflects both technical and clinical considerations. BIA, recommended for longitudinal body composition monitoring, requires patient cooperation generally achievable from four years of age, with substantially reduced reliability below this threshold. Additionally, the participating centers’ clinical experience is concentrated in patients aged four years and above, and nutritional assessment tools validated for infants and toddlers, including WHO weight-for-length indices, head circumference monitoring, and age-specific growth velocity assessment, differ from the anthropometric and body composition approaches described in this document. The distinct metabolic demands and feeding requirements of younger children warrant a dedicated consensus process. Clinicians managing patients below four years are directed to WHO growth standards and ESPGHAN enteral nutrition recommendations for infants and young children.

Finally, as with all consensus-based recommendations, these are time-dependent. Pediatric oncology nutrition is an evolving field, with ongoing research in areas such as gut microbiome modulation, postbiotic and prebiotic interventions, advances in body composition assessment, and the nutritional impact of newer therapies, including immunotherapy and targeted treatments. Our recommendations reflect the best available evidence and expert opinion at the time they were developed and will need to be revisited as new data emerge. Future updates should aim to incorporate a more formal evidence review process and a structured plan for periodic revision.

## 5. Conclusions

This consensus document provides a practical and standardized framework for nutritional intervention in pediatric oncology. Consistent implementation of these recommendations has the potential to improve nutritional status, treatment tolerance, and overall quality of life in pediatric oncology patients. The document is intended to remain dynamic and open to periodic updates as new scientific evidence emerges.

## Figures and Tables

**Table 1:** Calorie intake values based on age, sex and body weight. (Values represent Estimated Average Requirements (EARs) for normal physical activity).

Age (Years)	Boys (Kcal/kg/Day)	Girls (Kcal/kg/Day)
4–5	77	74
5–6	74.5	71.5
6–7	72.5	69.5
7–8	70.5	66.5
8–9	68.5	64
9–10	66.5	61
10–11	64.5	58
11–12	62.5	55
12–13	60	52
13–14	58	49.5
14–15	55.5	47
15–16	53.5	45.5
16–17	51.5	44.5
17–18	50.5	44

**Table 2:** Caloric intake for healthy preschool- and school-aged children. (Values represent EARs for normal physical activity).

Age (Years)	4–5	6–9	10–13 (Boys)	10–13 (Girls)
Energy (Kcal/day)	1600	1900	2250	2100

**Table 3:** Estimated daily protein requirements in healthy children and adolescents.

Age Group	Reference Protein Intake (g/kg/Day)	Usual Clinical Range (g/kg/Day)
4–6 years	0.91	0.9–1
7–10 years	0.9	0.9–1
11–14 years (Boys)	0.91	0.9–1
11–14 years (Girls)	0.9	0.9–1
15–18 years (Boys) Normal physical activity	0.92	0.85–0.95
15–18 years (Girls) Normal physical activity	0.85	0.85–0.95
15–18 years (Boys) Intense physical activity	1.2	1–1.2
15–18 years (Girls) Intense physical activity	1	1–1.2

**Table 4:** Recommended daily protein intake for healthy children.

Age (Years)	4–5	6–9	10–13 (Boys)	10–13 (Girls)
Protein (g/day)	30	36	43	41

**Table 5:** Recommended daily micronutrient intakes by age and sex.

Age (Years)	4–5	6–9	10–13 (Boys)	10–13 (Girls)	Adolescents (Boys)	Adolescents (Girls)
B1 (mg)	0.7	0.8	0.9	0.9	1.2	1
B2 (mg)	0.9	1	1.4	1.3	1.3	1.1
B6 (mg)	0.9	1.1	1.2	1.1	1.4	1.2
B12 (mcg)	1.4	1.7	2.1	2.1	2.4	2.4
Niacin (mg)	11	13	15	14	16	14
Folate (mcg)	200	250	300	300	400	400
Vitamin C (mg)	55	55	60	60	75	65
Pantothenic ac (mg)	3	4	4	4	5	5
Biotin (mcg)	12	14	20	20	20	20
Vitamin A (mcg)	500	700	1000	800	900	700
Vitamin D (mcg)	15	15	15	15	15	15
Vitamin E (mg)	8	8	10	8	15	15
Vitamin K (mcg)	55	55	60	60	75	75
Calcium (mg)	800	800	1300	1300	1300	1300
Magnesium (mg)	130	180	250	240	410	360
Iron (mg)	10	10	12	15	11	15
Zinc (mg)	10	10	15	12	11	9
Iodine (mcg)	90	130	150	150	150	150
Fluoride (mg)	1	1.5	2	2	2	2
Selenium (mcg)	20	30	40	45	55	55

## Data Availability

The data supporting the findings of this study—including round-by-round agreement scores and a summary of statement modifications—are available in the Supplementary Material (Table S1). Individual panelist responses are not publicly available, as they were collected and stored anonymously and cannot be attributed to specific participants in accordance with the confidentiality commitments made to panel members prior to participation.

## Supplementary Materials

The following supporting information can be downloaded at: https://www.mdpi.com/article/10.3390/nu18121889/s1, Table S1: Delphi consensus results—percentage agreement per recommendation across Round 1 and Round 2; Table S2: Question and Answer format summary of consensus recommendations.

## Abbreviations

The following abbreviations are used in this manuscript:

BIA	Bioelectrical Impedance Analysis
BMI	Body Mass Index
cGVHD	Chronic Graft-Versus-Host Disease
COG	Children’s Oncology Group
e-Delphi	Electronic Delphi
EAR	Estimated Average Requirements
EFSA	European Food Safety Authority
EN	Enteral Nutrition
ESPEN	European Society for Clinical Nutrition and Metabolism
ESPGHAN	European Society for Paediatric Gastroenterology, Hepatology and Nutrition
FAO	Food and Agriculture Organization
GVHD	Graft-Versus-Host Disease
HSCT	Hematopoietic Stem Cell Transplantation
MCT	Medium-Chain Triglycerides
MUAC	Mid-Upper Arm Circumference
ONS	Oral Nutritional Supplements
PN	Parenteral Nutrition
PNST	Paediatric Nutritional Screening Tool
PYMS	Paediatric Yorkhill Malnutrition Score
SDM	Shared Decision-Making
SCAN	Nutrition Screening Tool for Childhood Cancer
SIOP	International Society of Paediatric Oncology
STAMP	Screening Tool for the Assessment of Malnutrition in Paediatrics
STRONGkids	Screening Tool for Risk on Nutritional Status and Growth
TSFT	Triceps Skinfold Thickness
WHO	World Health Organization
