# Supplementary material for: Consensus Recommendations for Nutritional Intervention in Pediatric Oncology (Ages 4–18 Years) on Behalf of the Romanian Society of Pediatric Hematology and Oncology and the Romanian Society of Pediatric Gastroenterology, Hepatology and Nutrition
Source: Nutrients. 2026 Jun 11;18(12):1889. doi: 10.3390/nu18121889 (PMC13305020; doi:10.3390/nu18121889)
Supplement: Supplementary file 1 [file nutrients-18-01889-s001.zip › nutrients-4348779-supplementary.pdf]

Supplementary Materials

Nutritional Management in Pediatric Oncology: A Multidisciplinary Delphi Consensus

Table S1. *Delphi consensus results—percentage agreement per recommendation across Round 1 and Round 2*

Rec. No.	Domain	Recommendation (abbreviated)	Round 1 agreement (%)	Mean score Round 1	Round 2 agreement (%)	Modified after Round 1	Key panel comments/reason for revision
**1**	Screening & Assessment	Systematic integration of clinical nutrition into standard management of pediatric ocology patients	**100%**	**9**	**-**	**No**	**-**
**2**	Screening & Assessment	Involvement of multidisciplinary team with expertise in pediatric oncology nutrition	**95.45%**	**8.45**	**-**	**No**	**-**
**3**	Screening & Assessment	Nutritional screening in all patients at time of diagnosis; comprehensive evaluation if positive screening or in the presence of major nutritional risk factors.	**100%**	**9**	**-**	**No**	**-**
**4**	Screening & Assessment	Nutritional screening at every hospital admission and at regular intervals throughout treatment, using validated tools such as STAMP, PYMS, PNST or STRONGkids	**100%**	**9**	**-**	**No**	**-**
**5**	Energy & Protein	Energy requirements estimated as comparable to healthy children of same age, sex and BMI	**100%**	**8.91**	**-**	**No**	**-**
**6**	Energy & Protein	Age-appropriate protein intake, with escalation up to 2.5 g/kg/day based on clinical context, in the absence of renal insufficiency.	**100%**	**9**	**-**	**No**	**-**

**7**	Energy & Protein	Balanced macronutrient distribution; increased lipid and reduced carbohydrate intake in insulin resistance	**100%**	**8.95**	**-**	**No**	**-**
**8**	Micronutrients	Vitamins and minerals at reference intake levels; targeted supplementation if deficiency documented	**100%**	**8.86**	**-**	**No**	**-**
**9**	Physical Activity	Daily physical activity in absence of absolute contraindications, tailored to age and clinical status	**100%**	**8.91**	**-**	**No**	**-**
**10**	Physical Activity	Supervised, age-appropriate resistance training in adolescents	**77.27%**	**8.27**	**100%**	**Yes**	Scope clarified following panel discussion to specify that resistance training applies to adolescents
**11**	Nutritional Interventions	Avoidance of energy-restricted diets in patients at nutritional risk	**100%**	**8.91**	**-**	**No**	**-**
**12**	Nutritional Interventions	Oral nutrition as first-line approach: counseling, dietary fortification, and oral nutritional supplements	**100%**	**9**	**-**	**No**	**-**
**13**	Nutritional Interventions	Omega-3 fatty acid supplementation in patients with progressive unintentional weight loss	**100%**	**8.82**	**-**	**No**	**-**
**14**	Nutritional Interventions	Proactive enteral nutrition strategy in patients at high nutritional risk with anticipated reduced intake	**100%**	**8.91**	**-**	**No**	**-**
**15**	Nutritional Interventions	Nasogastric tube as first-line enteral access; gastrostomy for prolonged support	**100%**	**8.86**	**-**	**No**	**-**

**16**	Nutritional Interventions	Initiation of enteral nutrition as continuous feeding; transition to bolus if tolerated	**100%**	**8.82**	**-**	**No**	**-**
**17**	Nutritional Interventions	Enteral formula selection according to gastrointestinal function and patient age	**100%**	**8.86**	**-**	**No**	**-**
**18**	Nutritional Interventions	Parenteral nutrition as rescue intervention when enteral nutrition is impossible or insufficient	**100%**	**8.91**	**-**	**No**	**-**
**19**	Nutritional Interventions	Gradual escalation of nutritional intake to prevent refeeding syndrome	**100%**	**8.86**	**-**	**No**	**-**
**20**	Chemotherapy	Adequate nutritional intake and age-appropriate physical activity throughout anticancer treatment	**100%**	**8.91**	**-**	**No**	**-**
**21**	Chemotherapy	Escalation of nutritional support from oral to enteral and parenteral according to tolerance	**100%**	**8.86**	**-**	**No**	**-**
**22**	HSCT	Intensive nutritional monitoring during intensive chemotherapy and HSCT	**100%**	**8.91**	**-**	**No**	**-**
**23**	HSCT	Enteral nutrition prioritized over parenteral when gastrointestinal tract is functional	**77.27%**	**8.18**	**100%**	**Yes**	Scope clarified following panel discussion to specify that enteral nutrition is prioritized over parenteral
**24**	HSCT	Glutamine supplementation may be considered in HSCT patients requiring parenteral nutrition	**90.91%**	**8.45**	**-**	**No**	**-**
**25**	HSCT	No routine low-bacterial-load diet; strict food hygiene and safe food-handling practices recommended	**77.27%**	**8.18**	**100%**	**Yes**	Statement revised following panel discussion to reflect current evidence, which does not support the routine use of low-bacterial-load

							diets beyond the first 30 days post-allogeneic HSCT
**26**	Radiotherapy	Adequate nutritional intake during radiotherapy to prevent deterioration and avoid treatment interruptions	**100%**	**8.91**	**-**	**No**	**-**
**27**	Radiotherapy	Early identification and prompt management of dysphagia	**100%**	**8.91**	**-**	**No**	**-**
**28**	Radiotherapy	Enteral nutrition preferred over parenteral in severe radiation-induced mucositis	**95.45%**	**8.68**	**-**	**No**	**-**
**29**	Radiotherapy	No routine glutamine supplementation or probiotics during radiotherapy	**100%**	**8.82**	**-**	**No**	**-**
**30**	Surgery	Specialized nutritional support before surgery, during hospitalization, and after discharge in patients at risk	**100%**	**8.86**	**-**	**No**	**-**
**31**	Surgery	7 –14 days preoperative nutritional support in malnourished patients undergoing major surgical procedures	**100%**	**8.91**	**-**	**No**	**-**
**32**	Surgery	Oral or enteral immunonutrition formulas in malnourished patients undergoing major oncologic surgery	**77.27%**	**8.18**	**100%**	**Yes**	Statement revised following panel discussion to restrict the recommendation to malnourished patients undergoing major oncologic surgery
**33**	Survivorship	Continued nutritional screening in cancer survivors, with comprehensive assessment in those at the risk.	**100%**	**8.95**	**-**	**No**	**-**

**34**	Survivorship	Regular physical activity adapted to age and physical status for cancer survivors	**77.27%**	**8.27**	**100%**	**Yes**	Statement revised following panel discussion to specify that physical activity recommendations for cancer survivors must be adapted to age and physical status, acknowledging the heterogeneity of this population in terms of treatment-related late effects, functional capacity, and residual toxicities.
**35**	Survivorship	Healthy diet and lifestyle: vegetables, fruits, whole grains, limited red meat and saturated fats for cancer survivors	**77.27%**	**8**	**100%**	**Yes**	Statement expanded following panel discussion to provide more specific dietary guidance for cancer survivors, including explicit mention of recommended food groups.
**36**	Survivorship	Lifestyle and dietary modifications initiated before completion of oncologic treatment	**100%**	**8.95**	**-**	**No**	**-**
**37**	Palliative Care	Nutritional screening continuation in patients receiving palliative care, with comprehensive assessment in those at the risk.	**100%**	**8.91**	**-**	**No**	**-**
**38**	Palliative Care	Nutritional interventions based on shared decision-making, considering prognosis and quality-of-life	**100%**	**8.86**	**-**	**No**	**-**
**39**	Palliative Care	Oral nutritional supplements and enteral nutrition preferred when clinical condition allows	**100%**	**8.82**	**-**	**No**	**-**
**40**	Palliative Care	Short-term limited parenteral hydration in dying phase to	**100%**	**8.77**	**-**	**No**	**-**

		exclude dehydration as precipitating factor					
**41**	HSTC	Butyrate supplementation in patients undergoing allogeneic HSCT, based on emerging evidence and limited clinical experience	**100%**	**9**	**-**	**No**	**-**

Legend:
Consensus threshold: ≥ 80% agreement, defined as ratings 7-9 on a 9-point Likert score (1= strongly disagree, 9= strongly agree)
Response rate: 22/22 (100%) both rounds.
Total recommendations: 41.

Table S2. *Question and Answer format summary of consensus recommendations*

No.	Question	Answer
**1**	Should clinical nutrition be integrated into the standard management of pediatric oncology patients?	Yes. Individualized nutritional assessment and support should be initiated at diagnosis and continued throughout treatment and survivorship
**2**	What type of team is recommended for nutritional screening and monitoring?	A team trained in pediatric oncology nutrition, including a pediatrician or pediatric gastroenterologist and a dietitian.
**3**	When and in whom should nutritional screening and assessment be performed?	In all pediatric oncology patients at the time of diagnosis with comprehensive evaluation for those with positive screening or major risk factors.
**4**	How often and with which tools should nutritional screening be repeated?	At each hospital admission and periodically throughout treatment and post-treatment follow-up, using validated pediatric tools such as STRONGkids, STAMP, PYMS, or PNST.

**5**	How should energy requirements be estimated if resting energy expenditure is not measured?	It should be estimated based on the requirements of healthy children of the same age, sex, and BMI, with individual adjustments as needed.
**6**	What is the recommended protein intake in patients without renal insufficiency?	A protein intake like that of healthy children, with the possibility of increasing it up to 2–2.5 g/kg/day, in absence of renal insuficiency.
**7**	How should macronutrient distribution be adapted in patients with insulin resistance?	By maintaining a balanced distribution while increasing lipid intake and reducing carbohydrate intake.
**8**	How should vitamins and minerals be provided?	In amounts close to the recommended dietary reference values, with targeted supplementation in cases of documented deficiency.
**9**	Is daily physical activity recommended for children with cancer?	Yes, in the absence of absolute contraindications, and it should be adapted to the patient's age and clinical status.
**10**	Is there a specific recommendation regarding physical activity for adolescents compared with younger children?	Yes. Adapted resistance exercises under specialized supervision are recommended for adolescents.
**11**	Are energy-restricted diets recommended in patients at nutritional risk?	No. Energy-restricted diets should be avoided.
**12**	What is the first-line nutritional intervention?	Oral nutritional intervention, including counseling, diet fortification, and oral nutritional supplements.
**13**	When is supplementation with omega-3 fatty acids recommended?	In patients with progressive, unintentional weight loss.
**14**	When is a proactive enteral nutrition strategy indicated?	In patients at high nutritional risk when a decrease in oral intake is anticipated.
**15**	What is the preferred access route for enteral nutrition?	A nasogastric tube; gastrostomy may be considered for long-term support.
**16**	How should enteral nutrition be initiated?	Initially as continuous feeding, with later transition to bolus feeding if tolerated.
**17**	What criteria guide the choice of enteral nutrition formula?	Gastrointestinal tract functionality and patient age.
**18**	When is parenteral nutrition indicated?	As a rescue intervention when enteral nutrition is not feasible or insufficient.
**19**	How can refeeding syndrome be prevented?	By gradually increasing nutritional intake.
**20**	How should nutritional status be maintained during active anticancer treatment?	By ensuring adequate nutritional intake and maintaining physical activity adapted to the patient's condition.

**21**	How should nutritional support be escalated according to tolerance?	From oral nutritional support to enteral and, if necessary, parenteral nutrition, depending on the patient's needs.
**22**	What type of nutritional monitoring is required in patients undergoing intensive chemotherapy or HSCT?	Intensive nutritional monitoring.
**23**	Which type of medical nutrition is preferred in HSCT patients?	Enteral nutrition whenever possible, provided that the gastrointestinal tract is functional.
**24**	Is glutamine supplementation recommended in HSCT patients?	It may be considered in patients requiring parenteral nutrition.
**25**	Are low-bacterial-load diets routinely recommended?	No. Strict adherence to food hygiene and safe food handling practices is recommended.
**26**	How should nutritional intake be managed during radiotherapy?	By maintaining adequate nutritional intake in order to prevent deterioration of nutritional status.
**27**	How should radiotherapy-induced dysphagia be addressed?	Through early detection and prompt management.
**28**	Which type of nutrition is preferred in severe radiation-induced mucositis?	Enteral nutrition, preferably over parenteral nutrition.
**29**	Is there sufficient evidence to support the use of glutamine or probiotics during radiotherapy?	No. There is insufficient high-quality evidence to support their routine use.
**30**	When is specialized nutritional support indicated in pediatric oncologic surgery?	Preoperatively, during hospitalization, and postoperatively in patients at nutritional risk or with malnutrition.
**31**	How long is preoperative nutritional support recommended?	For 7–14 days in malnourished patients undergoing major surgical procedures.
**32**	In which patients are perioperative immunonutrition formulas recommended?	In malnourished patients undergoing major oncologic surgical procedures.
**33**	Should nutritional screening continue in cancer survivors?	Yes, according to the same recommendations applied at the time of diagnosis.
**34**	What physical activity recommendations exist for survivors of pediatric cancer?	Regular physical activity and maintenance of an optimal body weight, adapted to age and physical status.
**35**	What nutritional recommendations exist for survivors of pediatric cancer?	A balanced diet appropriate for the survivorship phase, adapted to age and physical status.

**36**	What lifestyle recommendations exist for survivors of pediatric cancer?	Regular physical activity, maintenance of optimal body weight, and a healthy lifestyle adapted to age and physical status.
**37**	Should nutritional screening continue in palliative care?	Yes, according to the recommendation applied at the time of diagnosis.
**38**	How should decisions regarding nutritional interventions in palliative care be made?	Through shared decision-making, considering prognosis, expected benefits, and the potential burden of the intervention.
**39**	Are oral nutritional supplements and enteral nutrition recommended in patients receiving palliative care?	Yes, when the patient’s clinical condition allows.
**40**	Is the use of short and limited parenteral hydration recommended in patients in the terminal phase?	Yes, to exclude dehydration as a potential precipitating factor.
**41**	Is butyrate administration recommended after HSCT?	Yes, but it does not represent standard of care and requires individualized assessment.
